# Changes in Attitudes toward Tobacco Smoking and Factors Associated with Quitting in 9-Year Observation of PURE Poland Cohort Study

**DOI:** 10.3390/ijerph19116564

**Published:** 2022-05-27

**Authors:** Katarzyna Połtyn-Zaradna, Piotr Psikus, Katarzyna Zatońska

**Affiliations:** 1 Department of Population Health, Wroclaw Medical University, Bujwida St. 44, 50-345 Wrocław, Poland; katarzyna.zatonska@umw.edu.pl; 2 Calisia University, 62-800 Kalisz, Poland; burmistrz@um.kepno.pl

**Keywords:** tobacco smoking, cohort study, smoking cessation

## Abstract

(1) Background: This study aims to examine changes in tobacco smoking prevalence in the PURE Poland cohort study over the 9-year follow-up period. Moreover, it attempts to identify socio-demographic factors that affect changes in attitudes towards tobacco smoking. (2) Methods: The PURE Poland cohort study—baseline was performed in 2007–2010 and covered 2036 participants, including urban (59.4%) and rural (40.6%) residents of Lower Silesia, Poland. The following study reports the results of 1690 participants who took part in both the baseline (2007–2010) study and 9-year follow-up (2016–2019). (3) Results: There was a 3.5% decrease in current smokers during the analyzed period (from 20.2% at the baseline study to 16.7% in the 9-year follow-up). Living in rural area increased the likelihood of being a current smoker by more than 1.5-fold (OR = 1.65 CI = 1.26–2.14) and decreased the likelihood of being a former smoker (OR = 0.70 CI = 0.57–0.86). In the 9-year follow-up period, more women were current smokers than men (17.2% vs. 16.0%) and women had lower chances of being former smokers than men (OR = 0.77 CI = 0.62–0.95). People with a primary education had 1.5-fold higher likelihood of being a current smoker (OR = 1.45 CI = 1.03–2.05). Nearly 11% significant increase in the percentage of current smokers was observed in the oldest age group (1.9% in the baseline study vs. 12.6% in the follow-up period). (4) Conclusions: The results obtained during 9 years of observation indicate the necessity of intensifying anti-tobacco programs especially targeting women, elderly population, people with lower level of education, rural residents, and the unemployed.

## 1. Introduction

Tobacco smoking is one of the major modifiable predictors of premature mortality. According to the WHO, currently 1.3 billion people in the world smoke tobacco. Tobacco smoking causes more than eight million deaths each year. The reason for 7.2 million of these deaths is direct tobacco use, while approximately 1.2 million deaths are the result of passive smoking [1]. Globally, the WHO European Region has the highest adult smoking prevalence rate (28%), including one of the highest prevalence rates in women (19%) [1].

Since the 1990s, there has been a steady decline in the proportion of smokers in Poland. With the introduction of strong regulations in the 1990s, Poland was named a tobacco-control leader in Europe [2]. Over the period from 1990–1994, 51% of men and 25% of women smoked tobacco daily [3]. The WHO global report on trends in the prevalence of tobacco use from 2000–2025 estimates that the age-standardized proportion of current cigarette smokers in 2018 in Poland was 24.3% of all adults and 20.2% of adult women. On the contrary, in Iceland, which is a European country with the lowest percentage of current smokers, 11.0% of all adults and 11.4% of adult women smoked cigarettes [4]. Tobacco smoking remains a major public health issue in Poland, resulting in disability, reduced economic development, and premature mortality. According to the European Commission, approximately 50% of those who smoke tobacco die prematurely, resulting in an average loss of 14 life years [5]. Samet and Buran estimated that approximately 80,000 deaths in Poland were linked to tobacco smoking in 2017 [6]. According to a study on liability and the health costs of smoking, the estimated premature mortality costs in 2009 in Poland were EUR 56,183 million or 10.33% of GDP (gross domestic product) [7]. However, smoking cessation—especially before the age of 40—may dramatically reduce the risk of death and greatly improve quality of life. Therefore, it is critical to encourage all current smokers to quit smoking in order to reduce the disease burden and tobacco smoking-related mortality [8]. 

As tobacco smoking attitudes vary by age, sex, place of residence (urban–rural), and level of education and income [8,9,10,11,12,13,14,15], monitoring the tobacco smoking prevalence and initiating research concerning the determinants and consequences of tobacco smoking are essential for the development and implementation of effective health policy programs resulting in the reduction of tobacco smoking rates [9]. 

The PURE (Prospective Urban Rural Epidemiology) Poland study cohort, which was established in 2007–2010, has been the basis for unique nationwide studies concerning tobacco smoking patterns in urban (Wroclaw) and rural (Wroclaw area) populations. Our previous analyses revealed that tobacco smoking prevalence in the PURE Poland population only varies by age, place of residence, and level of education [15,16]. This study aims to examine changes in tobacco smoking prevalence in the PURE Poland cohort study over the 9-year follow-up period. Moreover, it attempts to identify socio-demographic factors that affect changes in attitudes towards tobacco smoking.

## 2. Materials and Methods

**Figure 1:**
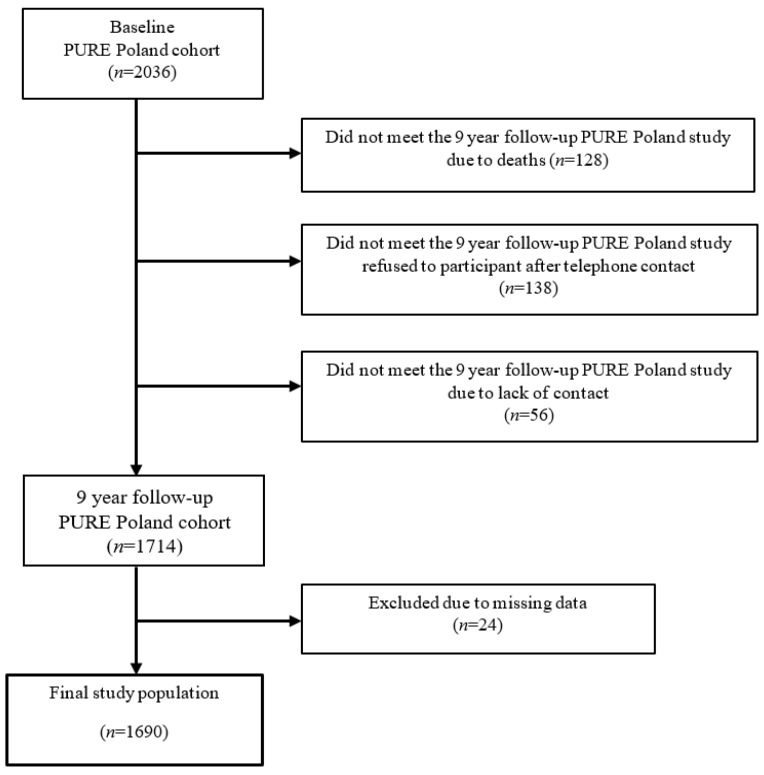
Flow chart of the Pure Poland study participants (*n* = 2036) and the final study population (*n* = 1690).

The PURE study includes 225,000 participants from 27 high-, middle-, and low-income countries [17]. The PURE Poland cohort study was performed in 2007–2010 and covered 2036 participants (1277 women and 758 men; age: 30–85 years), including urban (59.4%) and rural (40.6%) residents of Lower Silesia, Poland. The recruitment of cohort volunteers was announced in the Polish media (TV, radio, local press). Participants were selected to obtain a sample of the community that is as representative as possible [18,19]. All participants were assessed according to the PURE project protocol, which was described in detail elsewhere [18,19]. Every visit in the study center included a questionnaire study (individual health, family, household, food frequency, and international physical activity questionnaires), a blood draw, blood pressure measurement, and anthropometric measurements [18,19]. After the 9-year follow-up period, the re-contact rate was 84.2%. The following study reports the results of 1690 participants who took part in both the baseline study and 9-year follow-up (2016–2019). The flowchart diagram shows different stages of recruiting the final study population (Figure 1). Participants who died/resigned from the study between baseline and follow-up and who were not reached by phone were excluded from this analysis. If participants lacked any data regarding attitudes towards tobacco smoking, they were also excluded from the analysis. 

More than half of the participants were women, 64.8%, and urban residents, 59.4%. Participants were divided into three birth-year cohort: those born before 1940 (aged 67–85), 1940–1960 (aged 47–69) and 1961–1979 (aged 30–48). During the period covered by this study, the median age increased by 9 years and 75% of respondents were aged under 70. Detailed characteristics of the study population in the baseline study and after the 9-year follow-up are shown in Table 1.

The tobacco smoking history was assessed both in the baseline study and after the 9-year follow-up. The participants selected one of three possible responses: “I used tobacco products in the past”; “I currently use tobacco products” or “I have never used tobacco products”. Worldwide, specific criteria for the tobacco smoking history were added to the 9-year follow-up. In the baseline study, regular use of tobacco products was defined as consumption of at least one tobacco product per day. In the 9-year follow-up, current smokers were defined as participants who have smoked at least 100 cigarettes in their lifetime and currently smoke cigarettes daily or on certain days (non-daily). In addition, in the 9-year follow-up, never-smokers were defined as participants who have never smoked or who have smoked fewer than 100 cigarettes in their lifetime. Moreover, for the purpose of detailed analyses, 410 individuals who were former smokers on the first examination and on the final (ninth) examination were identified in the group of former smokers as “successful quitters”, whereas 84 individuals who were current smokers on the first examination and former smokers on the second examination were identified as “quitters”. The questionnaire regarding tobacco smoking referred to traditional tobacco products (cigarettes).

The data were analyzed using the chi-square test and logistic regression test, whereas the smoking status during the follow-up period was analyzed by sex, birth cohort, place of residence, and level of education. In detailed analyses, the following self-reported health problems were considered in the baseline study: smoking-related factors (such as cough for at least 2 weeks, wheezing or whistling in the chest, early morning cough with chest tightness), respiratory diseases (including chronic obstructive pulmonary disease (COPD), asthma, tuberculosis (TB)), cardiovascular diseases (CVD) (including cardiac infarction, coronary artery disease, heart failure, hypertension, and other heart diseases), and stroke. Furthermore, pack-years, presence of diabetes and hypertension, body weight, Alternate Healthy Eating index (AHEI) score, physical activity, and employment status were included in the analyses. Pack-years were calculated by multiplying the number of packs of cigarettes smoked per day by the number of years the person has smoked. The diabetes group consisted of individuals whose fasting plasma glucose was 126 mg/dL (7.0 mmol/L) or higher or those who self-reported diabetes diagnosis and diabetic treatment. The hypertension group consisted of individuals whose the average of three measurements met the ESC criteria (systolic blood pressure ≥ 140 and/or diastolic blood pressure ≥ 90 mmHg) or those who self-reported hypertension diagnosis and treatment. Patients were advised to sit quietly and rest for 5 min before blood pressure measurement. The appropriate cuff size was selected. The measurements were separated by 5-min breaks. Body mass was assessed using the body mass index (BMI). The participants were divided into four groups according to their BMI values: underweight (BMI < 18.5 kg/m^2^), normal body weight (BMI 18.5−24.99 kg/m^2^), overweight (BMI 25.00–29.99 kg/m^2^), and obesity (BMI ≥ 30.00 kg/m^2^). AHEI-2010 score was calculated according to methodology that was described by Chiuve et al. [20,21]. Physical activity was assessed using the International Questionnaire of Physical Activity (IPAQ)—long version [22].

The variables that were associated with current smoking at a significance level of *p*-value < 0.1 in the univariate analysis were included in multivariate modeling using the backward conditional regression method, where a two-sided *p*-value < 0.05 was considered statistically significant. The statistical analysis was performed using the STATISTICA software version 13.3 (TIBCO. Software Inc., Palo Alto, CA, USA)). The multivariate logistic regression analysis of the smoking status was performed using variables with *p*-value less than 0.05 in the univariate analysis. A backwards-stepwise method was applied using variables retained in the model if their logistic *p*-value likelihood ratio was less than 0.05. The combination of independent variables that gave the best explanation of the outcome (using the R2 statistics) was adopted.

Each participant gave informed consent to participate in the study. All human studies were reviewed by the appropriate bioethics committee, and they were conducted in accordance with the ethical standards contained in a relevant version of the 1964 Declaration of Helsinki (Positive opinion of the Bioethics Committee of the Wroclaw Medical University No. KB-443/2006).

## 3. Results

**Figure 2:**
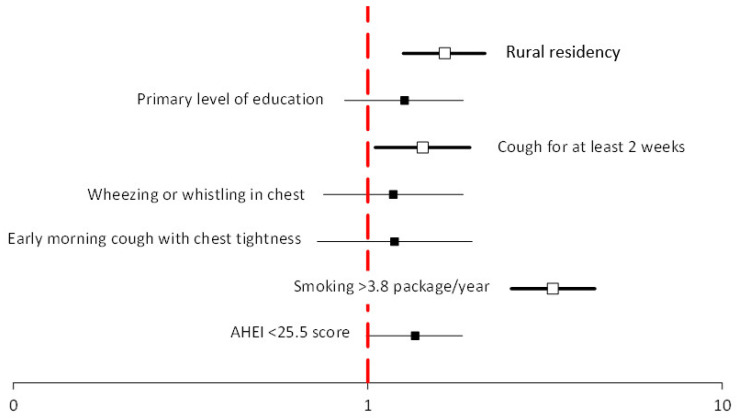
Multivariable logistic regression analysis of predictors of current smoking after the 9-year follow-up period.

**Figure 3:**
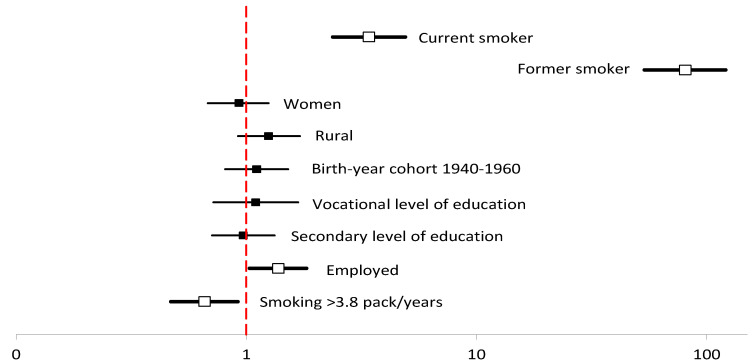
Multivariable logistic regression analyses of predictors of former smoking after the 9-year follow-up period.

There was a statistically significant, 3.5% decrease in current smokers during the period under analysis (from 20.2% in the baseline study to 16.7% in the 9-year follow-up period). In the baseline study, there were 20.2% current smokers, 31.5% former smokers, and 48.3% of never-smokers. After the 9-year follow-up period, the percentage of current smokers decreased to 16.7%, former smokers increased to 45.4%, and never-smokers decreased slightly to 47.9% (Table 2). Participants whose pack-years were ≥3.8 had over 3-fold higher risk (odds ratio (OR) = 3.36; confidence interval (CI) 2.59–4.40), and those who declared tobacco smoking in the baseline study were 75 times more likely to be current smokers in the 9-year follow-up period (OR = 75.2 CI = 51.5–110) (Table 3). Moreover, the likelihood was higher in participants who, in the baseline study, declared early morning cough with chest tightness, wheezing, or whistling in the chest or cough for at least 2 weeks (Table 3, Figure 2). In contrast, former smokers at the baseline study and the employed had a higher likelihood of being former smokers (Table 4, Figure 3). 

Attitudes towards tobacco smoking were statistically significantly differentiated by sex, birth cohort, place of residence, and level of education (Table 2). 

There was a statistically significant, 6.0%, decrease in the percentage of male current smokers from 22.0% in the baseline study to 16.0% in the follow-up period. Moreover, it should be noted that there was a statistically significant—8.4%—increase in the percentage of never smokers, resulting from the change in the definition of current smokers. This means that 50 male participants who declared in the baseline study to be never smokers smoked less than 100 cigarettes in their lifetime. For women, there was non-significant decrease in smoking prevalence (from 19.2% in the baseline study to 17.2% in the follow-up period). However, there was a significant 7.3% increase in the percentage of former smokers and a decrease in the percentage of never smokers by 5.3% (Table 2). This means that 58 women started tobacco smoking during the period under analysis and smoked at least 100 cigarettes and/or continued smoking. However, sex differences were not statistically significant (*p* >0.05). Sex was not a significant differentiator of being a current smoker (OR = 1.09 CI = 0.60–1.99) (Table 3). On the other hand, the 9-year follow-up revealed that being female reduces the likelihood of being a former smoker (OR = 0.77 CI = 0.62–0.95) (Table 4). In the multivariate regression analysis, sex was a non-significant factor of being a former smoker (Figure 3). 

Age is a significant differentiator of smoking patterns. In the baseline study, there was an increase in the percentage of current smokers as the age of the participants decreased. The highest percentage of current smokers (23.9%) was observed in the youngest individuals who were born in 1961–1979. In contrast, after the 9-year follow-up period, current smoking was the most prevalent in those born between 1940–1960 (17.3%). In terms of those who were born in 1961–1979, there was a significant 7.7% decrease in the percentage of current smokers (Table 2). Despite this, age was found to be a non-significant predictor of being a current smoker (OR = 0.70 CI = 0.39–1.28 in <1940 birth cohort and OR = 1.14 CI = 0.87–1.51 in 1940–1960 birth cohort) (Table 3). In contrast, those born in 1940–1960 are nearly one-third more likely to be a former smoker compared to others (OR = 1.27 CI 1.02–1.57) (Table 4).

A very disturbing, nearly 11% statistically significant increase in the percentage of current smokers was observed in the oldest age group (1.9% in the baseline study vs. 12.6% in the follow-up period). Because of the low size of the group of participants who were born before 1940, these results should be interpreted with caution (Table 2). 

The percentage of current smokers was higher in rural than urban residents (25.1% vs. 16.8% in the baseline study and 20.7 vs. 14.0% in the follow-up period, respectively). Only for rural residents was there a statistically significant increase in the percentage of former smokers (24.8% in the baseline study vs. 30.6% in the follow-up period) (Table 2). Living in rural area independently increased the likelihood of being a current smoker by more than 1.5 times (OR = 1.65 CI = 1.26–2.14) (Figure 2). In the univariate logistic regression analysis, rural residency decreased the likelihood of being a former smoker (OR = 0.70 CI = 0.57–0.86) (Table 4).

The lowest percentage of current smokers was found in the higher education group (15.1% in the baseline study vs. 12.6 in the follow-up period). Excluding those with a primary education, in whom the percentage of current smokers increased from 17.7% in the baseline study to 21.6% in the follow-up period, there was a decrease in current smokers in all other groups (Table 2). The level of education significantly affected the likelihood of being a current smoker or a former smoker. People with a primary education had nearly 1.5 times the likelihood of being current smokers (OR = 1.45 CI = 1.03–2.05) (Table 3). On the other hand, secondary education was a factor that increased the likelihood of being a former smoker (OR = 1.28 CI 1.04–1.56), whereas vocational education was a factor that decreased it (OR = 0.73 CI 0.55–0.97) (Table 4).

In the following section, the factors that affect successful smoking cessation or quitting smoking between baseline and follow-up are analyzed in detail. 

Baseline demographics for 459 former smokers on the first examination according to smoking status on the final examination conducted 9 years later are shown in Table 5. In general, participants who remained former smokers in the 9-year follow-up period (successful quitters) had a higher AHEI score, a lower MET (multiples of the resting metabolic rate) score for walking from place to place, a lower MET score for transportation-related physical activity, and they walked less during the day in their leisure time. 

**Figure 4:**
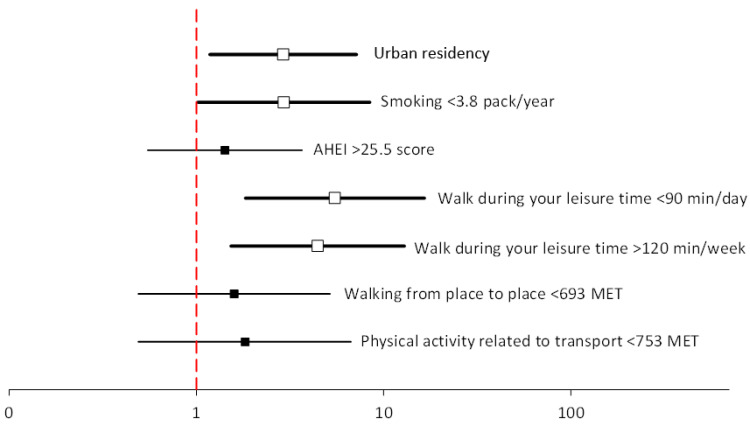
Multivariable logistic regression analyses of predictors of successful smoking cessation after the 9-year follow-up period.

The most significant predictors of successful smoking cessation included the amount of free time spent on walking, fewer pack-years (OR = 2.93; CI = 1.02–8.43), and living in an urban area (OR = 2.91; CI = 1.18–7.16) (Table 6, Figure 4). 

**Figure 5:**
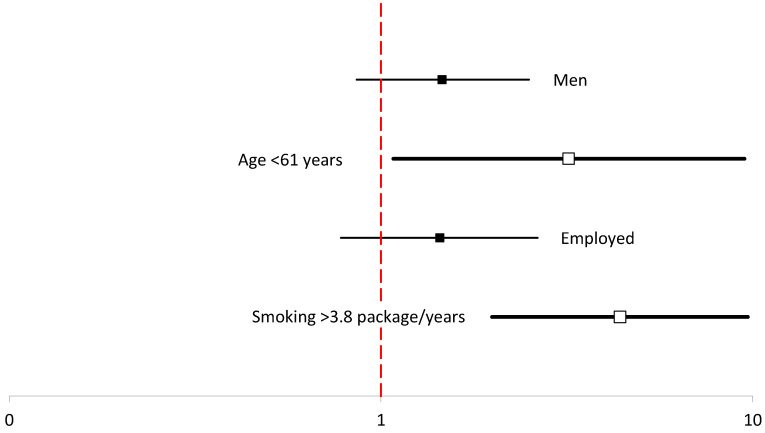
Multivariable logistic regression analyses of predictors of smoking cessation after the 9-year follow-up period.

In contrast, the most significant predictors of quitting smoking included the amount of free time, more pack-years (OR = 5.42 CI = 2.50–11.8), age > 61 years (OR = 5.03 CI = 1.75–14.2), status of being employed (OR = 2.20 CI = 1.27–3.79), and male sex (OR = 1.70 CI = 1.02–2.84) (Table 7, Figure 5).

## 4. Discussion

This study examines changes in tobacco smoking prevalence in the PURE Poland cohort study over the 9-year follow-up period. Moreover, it attempts to identify socio-demographic factors that affect changes in attitudes towards tobacco smoking. The results revealed a small but statistically significant decrease in the percentage of current smokers. There has been a systematic decrease in the percentage of current smokers in Poland since the 1990s [15,23,24,25]. The decrease was more observed in men; the youngest people, who were born in 1961–1979; and those with a vocational education. The reports published by the European Commission reveal that the percentage of current smokers across the EU decreased by 6.0% in 2006–2017 and by 2.0% in 2017–2020 [24,25]. In our cohort, the decrease was 3.5% in the 9-year follow-up period. Conforming to our results, other studies observed a similar decrease in tobacco smoking prevalence in Poland [14,26]. Several factors could have contributed to the overall decrease in smoking prevalence, including introduction of anti-tobacco governmental regulations [23] as well as increasing availability of cytisine, which increases the likelihood of smoking cessation [27,28]. An analysis of data published under the “Actual problems and events” study conducted by the Public Opinion Research Centre reveals that 21.8% of general Polish population smoked cigarettes in 2019 [29]. This percentage is higher than in our population (16.7%). The lower percentage of current smokers in this study compared to the general Polish population study was already observed in the baseline study (2007–2010). According to Global Adult Tobacco Survey (GATS) data, 30.3% of Polish population smoked cigarettes in 2010, whereas 20.2% of our cohort did at the time [30]. These differences may be due to discrepancies in age groups of participants. The GATS study included participants aged 15 and older, while our cohort includes individuals aged 30 and older. Moreover, Pesce et al. [10] revealed that in the 2000s, the peak in smoking prevalence in Europe was particularly pronounced in people around the age of 30 and in Eastern Europe in those aged 26. The impact of age differences in the study groups may be confirmed by the fact that at the beginning of our follow-up, there was a decrease in smoking prevalence with the increase of age. The highest percentage of current smokers was found in the youngest participants, who were born in 1961–1979.

Sex was a factor that significantly differentiated tobacco smoking prevalence in this study. In the baseline study, the percentage of current smokers was higher in men compared to women (22.0% vs. 19.2%), while after the 9-year follow-up period, more women were current smokers (17.2% vs. 16.0%). Other studies of the Polish population reveal higher smoking prevalence in men over the entire follow-up period [13,14,29,30]. The higher smoking prevalence in women in our study may be partially explained by the fact that our population is generally older than Polish population. Pinkas et al., observed the highest smoking prevalence in women aged 30–39 and 50–59 years [31]. Moreover, after the 9-year follow-up period, women who participated in our study were less likely to be former smokers than men (OR = 0.77 CI = 0.62–0.95), which is consistent with the results of other Polish study [14] (women: OR = 0.76Cl = 0.55–1.07 in urban area in 2012 and OR = 0.50 Cl = 0.35–0.72 in rural area in 2012). Furthermore, women were less likely to quit smoking during the follow-up period. Higher decline in smoking prevalence in men versus women has been already observed in Europe [32]. It has been estimated that smoking-attributable mortality in women may surpass smoking-attributable mortality in men, which indicates that women should be targeted in anti-tobacco campaigns and interventions [32]. On the other hand, there was a positive—more than 7%—increase in the percentage of ex-smokers in women (from 25.9% to 33.2%). The highest decline in the percentage of current smokers in study participants was observed in the youngest birth cohort—1961–1979 (from 23.9% to 16.2%). However, the 1940–1960 birth cohort participants were most likely to be former smokers over the 9-year follow-up period, whereas those aged under 61 were three times more likely to quit smoking during the follow-up period. These results correspond to the EU report’s results [25] which revealed that 22.0% of 40–54-year-olds and 30% of 55-year-olds and older quit smoking. 

In this study, rural residents were more likely to be current smokers both in the baseline study and during the 9-year follow-up period; however, this percentage decreased over the follow-up period in both areas (from 25.1% to 20.7% in rural area and from 16.8% to 14.0% in urban area). Living in rural area increased the likelihood of being a current smoker, decreased the likelihood of being a former smoker, and decreased the likelihood of successful smoking cessation after the 9-year follow-up period by more than two times. Our results correspond to those obtained by Sozańska et al.’s [14] study conducted in the same region of Poland. On the other hand, studies including the general Polish population in the same period of time as our follow-up reveal that urban residents are more frequently current smokers than rural residents [29,30]. The differences observed in terms of tobacco smoking attitudes between urban and rural residents may indicate regional variations within the Polish population. Other global studies also found a higher percentage of current smokers in rural residents [33,34].

According to our results, tobacco smoking attitudes vary by the level of education. The percentage of current smokers both in the baseline study and over the 9-year follow-up period decreased as the level of education increased. A secondary education increased the likelihood of being a former smoker nearly 1.3-fold, while a vocational education decreased this likelihood by nearly one-third. Moreover, it should be noted that during the follow-up period, participants with a primary education were least likely to quit smoking. The obtained results are consistent with the results of studies conducted in the general Polish population and other studies according to which smoking prevalence was higher in individuals with a lower education [24,25,35,36]. In both 2010 and 2019, individuals with a higher education were the smallest group of current smokers in Poland (24.5% and 8.3%, respectively) [29,30]. Women with a secondary education had two times higher likelihood of being former smokers (OR = 2.01 CI = 1.15–3.32), whereas men with a higher education had more than four times higher likelihood of being former smokers (OR = 4.22 CI = 1.67–10.66) [29].

In addition, we searched for other predictors that affect tobacco smoking attitudes in the study population over the 9-year follow-up period. The most significant factors that were associated with being current smoker, in addition to living in rural area, included a higher number of pack-years and a smoking-related factor, such as cough for at least 2 weeks. In contrast, factors that increased the likelihood of maintaining the status of a former smoker (successful quitter), in addition to living in urban area, were associated with fewer pack-years and amount of free time spent on walking (walking during leisure time >120 min per week and <90 min per day). Factors that increased the likelihood of smoking cessation during 9 years of observation in our cohort, in addition to male sex, were more pack-years, age <61 years, and being employed. The Polish population studies described by Kaleta at al. [13], as well as other studies, indicate a positive effect of being employed on tobacco smoking attitudes. According to other studies, unemployed men smoked cigarettes significantly more frequently on a daily basis than employed ones (OR = 1.8 CI = 1.4–2.4) [13,25,36,37]. In contrast, diagnosis of respiratory diseases, CVD, stroke, diabetes, or hypertension in the baseline study had no effect on smoking prevalence. An analogous lack of correlation was found by Tonnesen at al., following a 10-year follow-up of 12,283 participants in the Copenhagen General Population Study [38]. 

An interesting observation from our study concerns the increase in the prevalence of current smokers in the oldest age group. This birth cohort was small, and therefore, the conclusions should be interpreted with caution. This observation draws attention to the fact that elderly population should not be omitted in the anti-tobacco preventive programs. The elderly population may still believe that if they continued to smoke throughout their life, there is no point is quitting at their current age, whereas smoking cessation at any age decreases the risk of premature mortality and should be strongly encouraged [39,40]. Considering demographic changes towards the aging population, there is an urgent need to target the elderly with the anti-tobacco campaigns. 

When relating the results of our study to the general population, some limitations must be taken into consideration, e.g., the lack of biochemical verification of non-smoker status. According to West et al.’s findings, this may contribute to approximately 4% lower rates of tobacco smoking prevalence [41]. On the other hand, this was a study that did not focus on tobacco smoking attitudes but on many other health parameters; the study participants did not have strong reasons to not declare their actual smoking status. Another factor is the limited size of the group of oldest participants (birth cohort <1940, *n* = 103) that limits drawing inferences. Due to the global PURE study design, the population <30 years of age was not analyzed. The analyses did not include the income level that is considered to be one of the factors that affect tobacco smoking attitudes [42]. However, the level of education (primary, vocational, secondary, higher) was analyzed in detail and professional activity was taken into consideration, which correlates to a large extent with the level of income earned in Poland. The questionnaires in the PURE study focused on traditional tobacco products (cigarettes) and lacked questions regarding the electronic cigarettes or vaping. As the use of these products gains popularity, it is one of the limitations of our study. Although we observe the changes in attitudes toward tobacco smoking, the study did not investigate the methods of smoking cessation (e.g., pharmacotherapy), so we cannot draw conclusions on the most successful methods. 

On the other hand, the study strength is that it is a longitudinal cohort study. The application of identical methodology at each follow-up makes it possible to infer changes in tobacco smoking attitudes over the long-term follow-up of the same individuals at different stages of their life.

## 5. Conclusions

The results obtained during the 9-year follow-up indicate the necessity of intensifying anti-tobacco programs especially targeting women, less educated people, rural residents, the unemployed, and the elderly population. Preventive programs should also be directed towards former smokers to encourage them to maintain their status since our observations indicate that some participants relapsed over time.

## Figures and Tables

**Table 1:** Characteristics of 1690 participants of the PURE Poland study.

Sex	Total *n* = 1690		Urban *n* = 1004		Rural *n* = 686	
Man *n* (%)	595 (35.2%)		366 (36.5%)		229 (33.4%)	
Women *n* (%)	1095 (64.8%)		638 (63.5%)		457 (66.6%)	
	Baseline 2007–2010	9-year study	Baseline 2007–2010	9-year study	Baseline 2007–2010	9-year study
Man						
Age: median (range)	55 (45–61)	64 (54–70)	55 (47–61)	64 (56–70)	53 (45–61)	62 (54–70)
Birth-year cohort						
<1940	35 (5.9%)		13 (3.6%)		22 (9.6%)	
1940–1960	379 (63.7%)		251 (68.6%)		128 (55.9%)	
1961–1979	181 (30.4%)		102 (27.8%)		79 (34.5%)	
Women						
Age: median (range)	55 (48–61)	64 (57–70)	55 (49–60)	64 (58–69)	53 (47–61)	62 (56–70)
Birth-year cohort						
<1940	68 (6.2%)		21 (3.3%)		47 (10.3%)	
1940–1960	757 (69.1%)		478 (74.9%)		279 (61.1%)	
>1960	270 (24.7%)		139 (21.8%)		131 (28.7%)	
Total						
Age: median (Q1–Q3)	55 (47–61)	64 (56–70)	55 (49–61)	64 (58–70)	53 (46–61)	62 (55–70)
Birth-year cohort						
<1940	103 (6.1%)		34 (3.4%)		69 (10.1%)	
1940–1960	1136 (67.2%)		729 (72.6%)		407 (59.3%)	
>1960	451 (26.7%)		241 (24.0%)		210 (30.6%)	
Education level						
Primary	232 (13.7%)		34 (3.4%)		198 (28.9%)	
Vocational	267 (15.8%)		59 (5.9%)		208 (30.3%)	
Secondary	675 (40,0%)		448 (44.6%)		227 (33.1%)	
Higher	516 (30.5%)		463 (46.1%)		53 (7.7%)	

**Table 2:** General characteristics of attitudes towards tobacco smoking in 1690 participants of the PURE Poland study.

Characteristics	Ever Smokers								Never Smokers		*p*-Value					
	Current Smokers			Former Smokers			Total									
	Baseline 2007–2010	9-Year Study	*p*-Value	Baseline 2007–2010	9-Year Study	*p*-Value	Baseline 2007–2010	9-Year Study	Baseline 2007–2010	9-Year Study	(a)	(b)	(c)	(d)	(e)	(f)
Total *n* = 1690 % (95% Cl)	341 20.2 (18.3–22.1)	283 16.7 (15.0–18.5)	0.010	532 31.5 (29.3–33.7)	598 35.4 (33.1–37.7)	0.018	873 51.7 (49.3–54.0)	881 52.1 (49.7–54.5)	817 48.3 (46.0–50.7)	809 47.9 (45.5–50.3)	-	-	0.010	0.810	-	-
Sex																
Men *n* = 595 % (95% Cl)	131 22.0 (18.7–25.3)	95 16.0 (13.0–18.9)	0.013	248 41.7 (37.7–45.6)	234 39.3 (35.4–43.3)	0.484	379 63.7 (59.8–67.6)	329 55.3 (51.3–59.3)	216 36.3 (32.4–40.2)	266 44.7 (40.7–48.7)	<0.001	<0.001	0.004	0.004	0.043	0.062
Women *n* =1095 % (95% Cl)	210 19.2 (16.8–21.5)	188 17.2 (14.9–19.4)	0.263	284 25.9 (23.3–28.5)	364 33.2 (30.5–36.0)	0.001	494 45.1 (42.2–48.1)	552 50.4 (47.4–53.4)	601 54.9 (51.9–57.8)	543 49.6 (46.6–52.6)			<0.001	0.015		
Birth-year cohort																
<1940 *n* = 103 % (95% Cl)	2 1.9 (0.0–4.6)	13 12.6 (6.2–19.0)	0.005	31 30.1 (21.2–39.0)	33 32.0 (23.0–41.1)	0.764	33 32.0 (23.0–41.1)	46 44.7 (35.1–54.3)	70 68.0 (58.9–77.0)	57 55.4 (45.7–64.9)	<0.001	<0.001	0.009	0.086	0.004	0.018
1940–1960 *n* = 1136 % (95% Cl)	231 20.3 (18.0–22.7)	197 17.3 (15.1–19.5)	0.090	391 34.4 (31.7–37.2)	422 37.1 (34.3–40.0)	<0.001	622 54.8 (51.9–57.6)	619 54.5 (51.6–57.4)	514 45.2 (42.4–48.1)	517 45.5 (42.6–48.4)			<0.001	0.902		
1961–1979 *n* = 451 % (95% Cl)	108 23.9 (20.0–27.9)	73 16.2 (12.8–19.6)	0.008	108 23.9 (20.0–27.9)	143 31.7 (27.4–36.0)	<0.001	216 47.9 (43.3–52.5)	216 47.9 (43.3–52.5)	233 51.7 (47.1–56.3)	235 52.1 (47.5–56.7)			<0.001	0.998		
Place of residence																
Urban *n* = 1004 % (95% Cl)	169 16.8 (14.5–19.1)	141 14.0 (11.9–16.2)	0.098	362 36.1 (33.1–39.0)	388 38.6 (35.6–41.7)	0.281	531 52.9 (49.8–56.0)	529 52.7 (49.6–55.8)	473 47.1 (44.0–50.2)	475 47.3 (44.2–50.4)	<0.001	0.240	0.180	0.964	<0.001	0.612
Rural *n* = 686 % (95% Cl)	172 25.1 (21.8–28.3)	142 20.7 (17.7–23.7)	0.081	170 24.8 (21.6–28.0)	210 30.6 (27.2–34.1)	0.039	342 49.9 (46.1–53.6)	352 51.3 (47.6–55.1)	344 50.1 (46.4–53.9)	334 48.7 (44.9–52.4)			0.027	0.627		
Level of Education																
Primary *n* = 232 % (95% Cl)	41 17.7 (12.8–22.6)	50 21.6 (16.3–26.8)	0.332	61 26.3 (20.6–32.0)	76 32.8 (26.7–38.8)	0.215	102 44.0 (37.6–50.4)	126 54.3 (47.9–60.7)	130 56.0 (49.6–62.4)	106 45.7 (39.3–52.1)	<0.001	<0.001	0.083	0.033	0.002	0.013
Vocational *n* = 267 % (95% Cl)	78 29.2 (23.8–34.7)	50 18.7 (14.0–23.4)	0.019	69 25.8 (20.6–31.1)	79 29.6 (24.1–35.1)	0.443	147 55.1 (49.1–61.0)	129 48.3 (42.3–54.3)	120 44.9 (39.0–50.9)	138 51.7 (45.7–57.7)			0.018	0.141		
Secondary *n* = 675 % (95% Cl)	144 21.3 (18.2–24.4)	118 17.5 (14.6–20.3)	0.117	247 36.6 (33.0–40.2)	262 38.8 (35.1–42.5)	0.494	391 57.9 (54.2–61.7)	380 56.3 (52.6–60.0)	284 42.1 (38.3–45.8)	295 43.7 (40.0–47.4)			0.199	0.582		
Higher *n* = 516 % (95% Cl)	78 15.1 (12.0–18.2)	65 12.6 (9.7–15.5)	0.283	155 30.0 (26.1–34.0)	181 35.1 (31.0–39.2)	0.162	233 45.2 (40.9–49.4)	246 47.7 (43.4–52.0)	283 54.8 (50.6–59.1)	270 52.3 (48.0–56.6)			0.174	0.454		

(a) Chi-square test comparing current smokers, former smokers, and never smokers at baseline 2007; (b) chi-square test comparing ever smokers and never smokers at baseline 2007–2010; (c) chi-square test comparing current smokers, former smokers, and never smokers at baseline 2007–2010 and 9-year study; (d) chi-square test comparing ever smokers and never smokers at baseline 2007–2010 and 9-year study; (e) chi-square test comparing current smokers, former smokers, and never smokers in 9-year study; (f) chi-square test comparing ever smokers and never smokers in 9-year study.

**Table 3:** Odds ratios and 95% confidence intervals of predictors of current smoking after the 9-year follow-up period.

Parameters	Current Smokers *CS*_9*y*_ (9-Year Study)		Chi-Square Test: *p*-Value	OR (95% CI) *
	Yes *n* = 283	No *n* = 1407		
Current smoker (baseline):			<0.001	
Yes	234 (82.7%)	84 (6.0%)		**75.2 (51.5–110)**
No	49 (17.3%)	1323 (94.0%)		1.00 (ref.)
Former smoker (baseline):			<0.001	
Yes	49 (17.3%)	410 (29.1%)		**0.51 (0.37–0.71)**
No	234 (82.7%)	997 (70.9%)		1.00 (ref.)
Sex:			0.573	
Women	188 (66.4%)	907 (64.5%)		**1.09 (0.83–1.43)**
Men	95 (33.6%)	500 (35.5%)		1.00 (ref.)
Place of residence:			<0.001	
Rural	142 (50.2%)	544 (38.7%)		**1.60 (1.24–2.06)**
Urban	141 (49.8%)	863 (61.3%)		1.00 (ref.)
Birth-year cohort <1940:			0.307	
Yes	13 (4.6%)	90 (6.4%)		0.70 (0.39–1.28)
No	270 (95.4%)	1317 (93.6%)		1.00 (ref.)
Birth-year cohort 1941–1960:			0.384	
Yes	197 (69.6%)	939 (66.7%)		1.14 (0.87–1.51)
No	86 (30.4%)	468 (33.3%)		1.00 (ref.)
Primary level of education:			0.040	
Yes	50 (17.7%)	181 (12.9%)		**1.45 (1.03–2.05)**
No	233 (82.3%)	1226 (87.1%)		1.00 (ref.)
Vocational level of education:			0.392	
Yes	50 (17.7%)	217 (15.4%)		1.18 (0.84–1.65)
No	233 (82.3%)	1190 (84.6%)		1.00 (ref.)
Secondary level of education:			0.567	
Yes	118 (41.7%)	558 (39.7%)		1.09 (0.84–1.41)
No	165 (58.3%)	849 (60.3%)		1.00 (ref.)
Cough for at least 2 weeks:			0.009	
Yes	74 (26.2%)	269 (19.1%)		**1.50 (1.11–2.01)**
No	209 (73.8%)	1138 (80.9%)		1.00 (ref.)
Wheezing or whistling in the chest:			0.049	
Yes	35 (12.4%)	119 (8.5%)		**1.53 (1.02–2.28)**
No	248 (87.6%)	1288 (91.5%)		1.00 (ref.)
Early morning cough with chest tightness:			0.006	
Yes	30 (10.6%)	83 (5.9%)		**1.89 (1.22–2.93)**
No	253 (89.4%)	1324 (94.1%)		1.00 (ref.)
Respiratory diseases:			0.502	
Yes	14 (4.9%)	84 (6.0%)		0.82 (0.46–1.67)
No	269 (95.1%)	1323 (94.0%)		1.00 (ref.)
CVD:			0.074	
Yes	41 (14.5%)	267 (19.0%)		0.72 (0.51–1.03)
No	242 (85.5%)	1140 (81.0%)		1.00 (ref.)
Stroke:			0.256	
Yes	7 (2.5%)	19 (1.4%)		1.85 (0.77–4.45)
No	276 (97.5%)	1388 (98.6%)		1.00 (ref.)
Diabetes:			0.297	
Yes	22 (7.8%)	86 (6.1%)		1.29 (0.80–2.11)
No	261 (92.2%)	1321 (93.9%)		1.00 (ref.)
Hypertension:			0.888	
Yes	101 (35.7%)	496 (35.3%)		1.02 (0.78–1.33)
No	182 (64.3%)	911 (64.7%)		1.00 (ref.)
Overweight or obese:			0.311	
Yes	192 (67.8%)	997 (70.9%)		0.87 (0.66–1.14)
No	91 (32.2%)	410 (29.1%)		1.00 (ref.)
Employed:			0.560	
Yes	157 (55.5%)	807 (57.4%)		0.93 (0.72–1.20)
No	126 (44.5%)	600 (42.6%)		1.00 (ref.)
Smoking pack years:			<0.001	
≥3.8	190 (67.1%)	532 (37.8%)		**3.36 (2.56–4.40)**
<3.8	93 (32.9%)	875 (62.2%)		1.00 (ref.)

* Bold values are statistically significant.

**Table 4:** Odds ratios and 95% confidence intervals of predictors of former smoking after the 9-year follow-up period.

Parameters	Former Smokers *FS*_9*y*_		Chi-Square Test: *p*-Value	OR (95% CI) *
	Yes *n* = 598	No *n* = 1092		
Current smoker (baseline):			<0.001	
Yes	84 (14.1%)	234 (21.4%)		**0.60 (0.46–0.79)**
No	514 (85.9%)	858 (78.6%)		1.00 (ref.)
Former smoker (baseline):			<0.001	
Yes	410 (68.6%)	49 (4.5%)		**46.4 (33.2–64.9)**
No	188 (31.4%)	1043 (95.5%)		1.00 (ref.)
Sex:			0.014	
Women	364 (60.9%)	731 (66.9%)		**0.77 (0.62–0.95)**
Men	234 (39.1%)	361 (33.1%)		1.00 (ref.)
Place of residence:			<0.001	
Rural	210 (35.1%)	476 (43.6%)		**0.70 (0.57–0.86)**
Urban	388 (64.9%)	616 (56.4%)		1.00 (ref.)
Birth-year cohort <1940:			0.531	
Yes	33 (5.5%)	70 (6.4%)		0.85 (0.56–1.31)
No	565 (94.5%)	1022 (93.6%)		1.00 (ref.)
Birth-year cohort 1940–1960:			0.034	
Yes	422 (70.6%)	714 (65.4%)		**1.27 (1.02–1.57)**
No	176 (29.4%)	378 (34.6%)		1.00 (ref.)
Primary level of education:			0.438	
Yes	76 (12.7%)	155 (14.2%)		0.88 (0.66–1.18)
No	522 (87.3%)	937 (85.8%)		1.00 (ref.)
Vocational level of education:			0.037	
Yes	79 (13.2%)	188 (17.2%)		**0.73 (0.55–0.97)**
No	519 (86.8%)	904 (82.8%)		1.00 (ref.)
Secondary level of education:			0.021	
Yes	262 (43.8%)	414 (37.9%)		**1.28 (1.04–1.56)**
No	336 (56.2%)	678 (62.1%)		1.00 (ref.)
Employed:			0.012	
Yes	366 (61.2%)	598 (54.8%)		**1.30 (1.06–1.60)**
No	232 (38.8%)	494 (45.2%)		1.00 (ref.)
Cough for at least 2 weeks:			0.236	
Yes	112 (18.7%)	231 (21.2%)		0.86 (0.67–1.10)
No	486 (81.3%)	861 (78.8%)		1.00 (ref.)
Wheezing or whistling in the chest:			0.250	
Yes	61 (10.2%)	93 (8.5%)		1.22 (0.87–1.71)
No	537 (89.8%)	999 (91.5%)		1.00 (ref.)
Early morning cough with chest tightness:			0.841	
Yes	39 (6.5%)	74 (6.8%)		0.96 (0.64–1.43)
No	559 (93.5%)	1018 (93.2%)		1.00 (ref.)
Respiratory diseases:			0.944	
Yes	35 (5.9%)	63 (5.8%)		1.02 (0.66–1.55)
No	563 (94.1%)	1029 (94.2%)		1.00 (ref.)
CVD:			0.147	
Yes	120 (20.1%)	188 (17.2%)		1.21 (0.94–1.56)
No	478 (79.9%)	904 (82.8%)		1.00 (ref.)
Stroke:			0.901	
Yes	9 (1.5%)	17 (1.6%)		0.97 (0.43–2.18)
No	589 (98.5%)	1075 (98.4%)		1.00 (ref.)
Diabetes:			0.504	
Yes	35 (5.9%)	73 (6.7%)		0.87 (0.57–1.32)
No	563 (94.1%)	1019 (93.3%)		1.00 (ref.)
Hypertension:			0.143	
Yes	225 (37.6%)	372 (34.1%)		1.17 (0.95–1.44)
No	373 (62.4%)	720 (65.9%)		1.00 (ref.)
Overweight or obese:			0.252	
Yes	431 (72.1%)	758 (69.4%)		1.14 (0.91–1.42)
No	167 (27.9%)	334 (30.6%)		1.00 (ref.)
Smoking pack years:			<0.001	
>3.8	337 (56.4%)	385 (35.3%)		**2.37 (1.93–2.91)**
<3.8	261 (43.6%)	707 (64.7%)		1.00 (ref.)

* Bold values OR are statistically significant.

**Table 5:** Baseline demographics of 459 former smokers on the first examination in 2007–2010 according to smoking status on the final examination in 2016–2019.

Parameters	Successfully Quit Smoking (9-Year Study)		*p*-Value
	Yes *n* = 410	No *n* = 49	
Age:			0.516
M ± SD	55.3 ± 8.9	54.5 ± 9.4	
Me (Q1; Q3)	56 (50; 61)	55 (48; 61)	
Min–Max	29–80	36–77	
Age of starting cigarette smoking:			0.344
M ± SD	19.4 ± 3.8	19.5 ± 4.2	
Me (Q1; Q3)	19 (18; 20)	20 (18; 20)	
Min–Max	10–47	8–40	
Smoking pack years:			0.501
M ± SD	14.0 ± 13.6	16.9 ± 17.4	
Me (Q1; Q3)	10 (5; 20)	10 (5; 24)	
Min–Max	0.1–100	0.1–72	
Average number of cigarettes smoked per day:			0.729
M ± SD	14.3 ± 9.4	15.4 ± 10.8	
Me (Q1; Q3)	11 (10; 20)	15 (8; 20)	
Min–Max	1–80	1–40	
Duration of cigarette smoking (years):			0.508
M ± SD	17.9 ± 10.4	18.7 ± 10.3	
Me (Q1; Q3)	18 (10; 25)	20 (10; 25)	
Min–Max	1–50	1–40	
SBP (mm Hg):			0.667
M ± SD	147 ± 21	145 ± 19	
Me (Q1; Q3)	144 (132; 158)	144 (130; 154)	
Min–Max	100–230	115–195	
DBP (mm Hg):			0.804
M ± SD	87 ± 11	86 ± 12	
Me (Q1; Q3)	86 (79; 93)	84 (78; 95)	
Min–Max	52–130	66–124	
Weight (kg):			0.968
M ± SD	78.5 ± 15.5	80.1 ± 18.8	
Me (Q1; Q3)	77 (67; 88)	75 (66; 92)	
Min–Max	48–136	54–130	
BMI (kg/m^2^):			0.763
M ± SD	28.6 ± 4.9	28.6 ± 5.8	
Me (Q1; Q3)	27.9 (24.9; 31.4)	27.8 (25.6; 30.1)	
Min–Max	19.1–46.7	17.9–47.0	
AHEI (score):			0.043
M ± SD	32.4 ± 7.5	30.2 ± 7.3	
Me (Q1; Q3)	31.5 (26.9; 37.5)	29.6 (24.9; 35.2)	
Min–Max	17.1–54.4	19.2–53.6	
Minutes/day you walk during your leisure time:			0.039
M ± SD	71.6 ± 57.8	113.4 ± 113.2	
Me (Q1; Q3)	60 (30; 90)	65 (40; 130)	
Min–Max	0–450	20–480	
MET score for walking to go from place to place:			0.047
M ± SD	614 ± 689	656 ± 534	
Me (Q1; Q3)	300 (120; 900)	600 (300; 900)	
Min–Max	0–3000	0–2160	
Transportation related physical activity MET score:			0.031
M ± SD	1270 ± 1480	1518 ± 1272	
Me (Q1; Q3)	765 (396; 1521)	1386 (594; 1848)	
Min–Max	0–9891	90–6030	

**Table 6:** Odds ratios and 95% confidence intervals of predictors of successful smoking cessation after the 9-year follow-up period.

Parameters	Successfully Quit Smoking (9-Year Study)		Chi-Square Test: *p*-Value	OR (95% CI) *
	Yes *n* = 410	No *n* = 49		
Sex:			0.775	
Women	243 (59.3%)	28 (57.1%)		1.09 (0.60–1.99)
Men	167 (40.7%)	21 (42.9%)		1.00 (ref.)
Place of residence:			0.005	
Urban	304 (74.1%)	27 (55.1%)		**2.34 (1.28–4.28)**
Rural	106 (25.9%)	22 (44.9%)		1.00 (ref.)
Age:			0.303	
≥56 years	216 (52.7%)	22 (44.9%)		1.37 (0.75–2.48)
<56 years	194 (47.3%)	27 (55.1%)		1.00 (ref.)
Primary level of education:			0.384	
Yes	45 (11.0%)	8 (16.3%)		0.63 (0.28–1.43)
No	365 (89.0%)	41 (83.7%)		1.00 (ref.)
Vocational level of education:			0.205	
Yes	43 (10.5%)	2 (4.1%)		2.75 (0.65–11.7)
No	367 (89.5%)	47 (95.9%)		1.00 (ref.)
Secondary level of education:			0.152	
Yes	190 (46.3%)	28 (57.1%)		0.65 (0.36–1.18)
No	220 (53.7%)	21 (42.9%)		1.00 (ref.)
Cough for at least 2 weeks:			0.538	
Yes	69 (16.8%)	6 (12.2%)		1.45 (0.59–3.54)
No	341 (83.2%)	43 (87.8%)		1.00 (ref.)
Wheezing or whistling in the chest:			0.606	
Yes	41 (10.0%)	3 (6.1%)		1.70 (0.51–5.72)
No	369 (90.0%)	46 (93.9%)		1.00 (ref.)
Early morning cough with chest tightness:			1.000	
Yes	19 (4.6%)	2 (4.1%)		1.14 (0.26–5.06)
No	391 (95.4%)	47 (95.9%)		1.00 (ref.)
Respiratory diseases:			1.000	
Yes	27 (6.6%)	3 (6.1%)		1.09 (0.32–3.70)
No	383 (93.4%)	46 (93.9%)		1.00 (ref.)
CVD:			0.702	
Yes	81 (19,8%)	8 (16,3%)		1.26 (0.57–2.80)
No	329 (80,2%)	41 (83,7%)		1.00 (ref.)
Stroke:			1.000	
Yes	8 (2,0%)	1 (2,0%)		0.96 (0.12–7.80)
No	402 (98,0%)	48 (98,0%)		1.00 (ref.)
Diabetes:			0.437	
Yes	26 (6.3%)	5 (10.2%)		0.60 (0.22–1.63)
No	384 (93.7%)	44 (89.8%)		1.00 (ref.)
Hypertension:			0.467	
Yes	162 (39.5%)	22 (44.9%)		0.80 (0.44–1.46)
No	248 (60.5%)	27 (55.1%)		1.00 (ref.)
Overweight or obese:			0.533	
Yes	301 (73.4%)	38 (77.6%)		0.80 (0.39–1.62)
No	109 (26.6%)	11 (22.4%)		1.00 (ref.)
Employed:			0.938	
Yes	245 (59.8%)	29 (59.2%)		1.02 (0.56–1.87)
No	165 (40.2%)	20 (40.8%)		1.00 (ref.)
Smoking pack years:			0.008	
<3.8	149 (36.3%)	8 (16.3%)		**2.93 (1.34–6.41)**
≥3.8	261 (63.7%)	41 (83.7%)		1.00 (ref.)
AHEI (score):			0.005	
≥25.5	331 (80.7%)	31 (63.3%)		**2.43 (1.30–4.57)**
<25.5	79 (19.3%)	18 (36.7%)		1.00 (ref.)
Walk during your leisure time:			0.015	
<90 min/day	193 (73.7%)	17 (53.1%)		**2.47 (1.17–5.21)**
≥90 min/day	69 (26.3%)	15 (46.9%)		1.00 (ref.)
Walk during your leisure time:			0.085	
≥120 min/week	179 (68.3%)	17 (53.1%)		1.90 (0.91–3.99)
<120 min/week	83 (31.7%)	15 (46.9%)		1.00 (ref.)
Walking from place to place:			0.033	
<693 MET	182 (55.3%)	15 (37.5%)		2.06 (1.05–4.06)
≥693 MET	147 (44.7%)	25 (62.5%)		1.00 (ref.)
Physical activity related to transport:			0.016	
<753 MET	171 (49.1%)	12 (29.3%)		2.33 (1.15–4.72)
≥753 MET	177 (50.9%)	29 (70.7%)		1.00 (ref.)

* Bold values OR are statistically significant.

**Table 7:** Odds ratios and 95% confidence intervals of predictors of smoking cessation after the 9-year follow-up period.

Parameters	Smoking Quitters *n* = 84	Continued Smokers *n* = 234	A vs. B *p*-Value	OR (95% CI) *
Sex:				
Men	37 (44.0%)	74 (31.6%)	0.040	**1.70 (1.02–2.84)**
Women	47 (56.0%)	160 (68.4%)		1.00 (ref.)
Age (years):				
< 61 years	80 (95.2%)	187 (79.9%)	0.001	**5.03 (1.75–14.2)**
≥ 61 years	4 (4.8%)	47 (20.1%)		1.00 (ref.)
Level of education:			0.112	
Primary	6 (7.1%)	42 (17.9%)		**0.35 (0.13–0.94)**
Vocational	39 (46.4%)	90 (38.5%)		1.06 (0.57–1.98)
Secondary	17 (20.3%)	48 (20.5%)		0.87 (0.41–1.83)
Higher	22 (26.2%)	54 (23.1%)		1.00 (ref.)
Cough for at least 2 weeks	21 (25.0%)	68 (29.1%)	0.477	0.81 (0.46–144)
Wheezing or whistling in the chest	14 (16.7%)	32 (13.7%)	0.504	1.26 (0.64–2.50)
Early morning cough with chest tightness	12 (14.3%)	28 (12.0%)	0.582	1.23 (0.59–2.54)
Respiratory diseases	2 (2.4%)	11 (4.7%)	0.526	0.49 (0.11–2.28)
CVD	15 (17.9%)	33 (14.1%)	0.410	1.32 (0.68–2.58)
Stroke	1 (1.2%)	6 (2.6%)	0.680	0.46 (0.05–3.86)
Diabetes	2 (2.4%)	17 (7.3%)	0.176	0.31 (0.07–1.37)
Hypertension	22 (26.2%)	79 (33.8%)	0.201	0.70 (0.40–1.21)
Overweight or obese	60 (71.4%)	154 (65.8%)	0.347	1.30 (0.75–2.24)
Employed	61 (72.6%)	128 (54.7%)	0.004	**2.20 (1.27–3.79)**
Smoking pack years ≥ 3.8	76 (90.5%)	149 (63.7%)	<0.001	**5.42 (2.50–11.8)**

* Bold values OR are statistically significant.

## Data Availability

The datasets used and/or analyzed during the current study are available from the corresponding author on reasonable request.
